# MR Imaging Characteristics of Solitary Fibrous Tumors of the Orbit: Case Series of 18 Patients

**DOI:** 10.1007/s00062-024-01400-8

**Published:** 2024-03-08

**Authors:** Christoph Ziegenfuß, Natalie van Landeghem, Chiara Meier, Roman Pförtner, Anja Eckstein, Philipp Dammann, Patrizia Haubold, Johannes Haubold, Michael Forsting, Cornelius Deuschl, Isabel Wanke, Yan Li

**Affiliations:** 1 Institute of Diagnostic and Interventional Radiology and Neuroradiology, University Hospital Essen, Hufelandstraße 55, 45147 Essen, Germany; 2 Department of Oral and Maxillofacial Surgery, University Hospital Essen, Kliniken-Essen-Mitte, Henricistraße 92, 45136 Essen, Germany; 3 Department of Ophthalmology, University Hospital Essen, Hufelandstraße 55, 45147 Essen, Germany; 4 Department of Neurosurgery and Spine Surgery, University Hospital Essen, Hufelandstraße 55, 45147 Essen, Germany; 5 Department of Diagnostic and Interventional Radiology, Kliniken Essen-Mitte, Henricistraße 92, 45136 Essen, Germany; 6 Swiss Neuroradiology Institute, Bürglistraße 29, 8002 Zürich, Switzerland

**Keywords:** Solitary fibrous tumor, SFT, MR orbit, Orbital tumor, MR imaging

## Abstract

**Purpose:**

Solitary fibrous tumor (SFT) of the orbit is a rare tumor that was first described in 1994. We aimed to investigate its imaging characteristics that may facilitate the differential diagnosis between SFT and other types of orbital tumors.

**Material and Methods:**

Magnetic resonance imaging (MRI) data of patients with immunohistochemically confirmed orbital SFT from 2002 to 2022 at a tertiary care center were retrospectively analyzed. Tumor location, size, morphological characteristics, and contrast enhancement features were evaluated.

**Results:**

Of the 18 eligible patients 10 were female (56%) with a mean age of 52 years. Most of the SFTs were oval-shaped (67%) with a sharp margin (83%). The most frequent locations were the laterocranial quadrant (44%), the extraconal space (67%) and the dorsal half of the orbit (67%). A flow void phenomenon was observed in nearly all cases (94%). On the T1-weighted imaging, tumor signal intensity (SI) was significantly lower than that of the retrobulbar fat and appeared predominantly equivalent (82%) to the temporomesial brain cortex, while on T2-weighted imaging its SI remained equivalent (50%) or slightly hyperintense to that of brain cortex. More than half of the lesions showed a homogeneous contrast enhancement pattern with a median SI increase of 2.2-fold compared to baseline precontrast imaging.

**Conclusion:**

The SFT represents a rare orbital tumor with several characteristic imaging features. It was mostly oval-shaped with a sharp margin and frequently localized in the extraconal space and dorsal half of the orbit. Flow voids indicating hypervascularization were the most common findings.

## Introduction

Solitary fibrous tumors (SFT) of the orbit are rare and diagnostically challenging neoplasms. The pleural manifestation of SFT was initially described in 1931 by Klemperer et al. [1] with its orbital occurrence reported for the first time in 1994 by Westra et al. [2]. Immunohistochemically, SFTs exhibit a characteristic positive expression pattern for CD34, CD99, and STAT6 [3]. The NAB2-STAT6 gene fusion is considered to be the underlying mutation leading to STAT6 overexpression, which is currently recognized as the primary event in disease development as well as a specific immunohistochemical marker for the diagnosis of SFT [4].

The exact incidence of orbital SFT remains unknown; however, there has been a notable increase in clinical reports recently, reflecting the growing interest of various clinical disciplines in this increasingly recognized tumor [5]. Primary orbital SFTs and secondary orbital involvement from those originating elsewhere were reported to account for less than 1% of all orbital space-occupying lesions [6]. The intermediate malignancy classification reflects the unpredictable nature of SFT, which can remain dormant for long periods of time or exhibit aggressive and metastatic behavior. Therefore, a better understanding of its clinical appearance and imaging characteristics can contribute to an early diagnosis and provide guidance for therapeutic decisions.

To date, only a limited number of retrospective studies about orbital SFT are available. Most have focused on histopathological findings or surgical management [7]. Imaging features of this rare tumor entity have been less frequently explored. Previously published investigations regarding imaging findings were case reports or series in relatively small patient collectives ranging from 1 to 15 individuals [8, 9]. Some of the suggested imaging techniques to distinguish SFTs from other orbital mass lesions, such as time-intensity curve of dynamic contrast-enhanced magnetic resonance (MR) imaging or MR-based radiomics nomogram, are difficult to apply in the routine radiology practice [10, 11]. Patients with clinical symptoms of orbital tumor usually undergo MR examination with available conventional imaging techniques at a primary hospital. Therefore, we aimed to investigate the imaging characteristics of orbital SFT with conventional MR imaging techniques and summarize the common imaging findings that could help radiologists to differentiate SFTs from other orbital tumors.

## Material and Methods

A retrospective analysis of patient data between 2002 and 2022 at a tertiary care center was performed. Ethical approval was obtained from the institutional review board (number 19-9091-BO), and our study was conducted in accordance with the Declaration of Helsinki. Inclusion criteria were as follows: immunohistochemical confirmation of SFT of the orbit on surgical specimens, available preoperative MR examination with sufficient diagnostic quality and patient age ≥ 18 years. Exclusion criteria were primary tumor location other than the orbit, such as sinonasal space with secondary orbital involvement and insufficient MR imaging quality.

Two radiologists (CZ with 1 year and YL with 14 years of neuroimaging experience) independently evaluated all imaging data. Discrepancies were resolved by consensus meeting. Most MR images were acquired with a 3 T MR scanner (Siemens, Skyra, Erlangen, Germany).

### Imaging Analysis

Demographic variables, such as gender and age of the patients at the time of surgical resection were documented. The exact tumor locations were classified by several categories: left or right side, intraconal or extraconal space, quadrant location, and ventral or dorsal half of the orbit. Morphological criteria included tumor shape, margin, signal homogeneity, and presence of flow voids. In addition, tumor size and volume were measured.

Mean signal intensity (SI) of the tumor was obtained by drawing a spherical region of interest on both T1-weighted and T2-weighted images and further compared with that of the retrobulbar fat tissue, temporal muscle, and brain cortex of the temporal lobe. For a rapid and simplified interpretation, the results were categorized as hypointense, isointense or hyperintense. Isointensity was determined when the SI difference between the aforementioned tissues and the SFT was less than 10% of the SI of SFT.

Tumor contrast uptake was analyzed on precontrast and postcontrast T1-weighted images. We determined the tumor contrast uptake ratio as follows: (SI of tumor on postcontrast T1 – SI of tumor on precontrast T1)/SI of tumor on precontrast T1. Additionally, the SI of the tumor was compared again with the SI of the brain cortex of the temporal lobe.

### Statistical Analysis

All results are presented as descriptive statistics. Continuous variables were reported as mean or median with interquartile range (IQR). Categorical variables were expressed as frequencies and percentages. Statistical analyses were performed using SPSS (version 29, IBM, Armonk, NY, USA).

## Results

Due to the rarity of the tumor, 21 patients from 2002 to 2022 were diagnosed with orbital SFTs by immunohistochemistry on surgical specimens. Of these, 18 met the inclusion criteria for the final analysis, 10 were female (56%) and the included patients had a mean age of 52 years (IQR 41–66 years).

### Tumor Location and Morphology

**Fig. 1:**
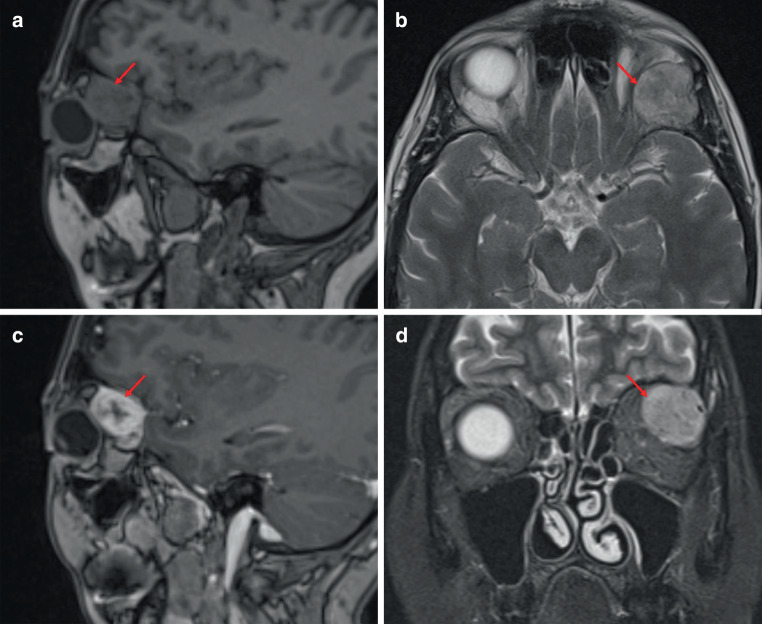
Magnetic resonance imaging of solitary fibrous tumor (SFT) in the laterocranial quadrant and extraconal space of the left orbit in a 57-year-old woman. **a** sagittal plane of non-contrast T1-weighted imaging with SFT (*red arrows in all images*), **b** axial plane of T2-weighted imaging, **c** sagittal plane of contrast-enhanced T1-weighted imaging, **d** coronal plane of STIR imaging. *SFT* solitary fibrous tumor, *STIR* short time inversion recovery

**Fig. 2:**
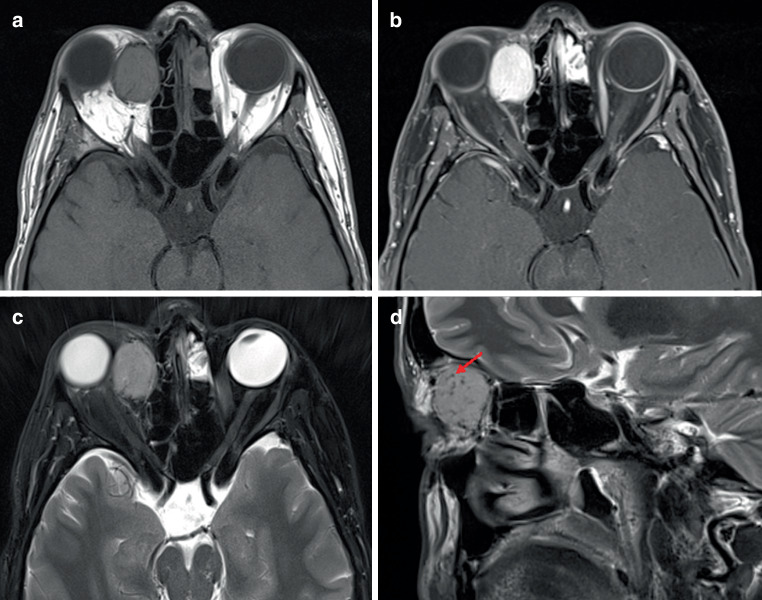
Magnetic resonance imaging of right-sided orbital solitary fibrous tumor (SFT) in a 29-year-old male. **a** axial plane of non-contrast T1-weighted imaging demonstrating similar signal intensity between SFT, brain cortex, and muscle temporalis, **b** axial plane of contrast-enhanced and fat-saturated T1-weighted imaging demonstrating markedly increased and homogeneous contrast uptake of the tumor, **c** axial plane of T2-weighted imaging with fat saturation showing similar signal intensity of the tumor and brain cortex, **d** sagittal plane of T2-weighted imaging demonstrating presence of tumor flow voids (*red arrow*) as a typical finding of SFT. *SFT* solitary fibrous tumor

All tumors were unilaterally located with a notable left-sided predominance accounting for 78% of cases. Predominant tumor locations were the laterocranial quadrant (44%), the extraconal space (67%), and the dorsal half of the orbit (67%) (Table 1 and Fig. 1). A significant proportion of these tumors exhibited an oval shape (67%) and well-defined margins (83%). The mean tumor volume was 7.9 cm^3^ with an IQR of 6.57–11.04 cm^3^, reflecting the variability in tumor size (median axial diameter 28 mm, IQR 22.75–34 mm, range 16–44 mm). Flow voids were observed in almost all cases (94%) (Table 2 and Fig. 2).

**Table 1:** Demographic characteristics of patients, tumor location and morphology

Categories	Variables	Total (*n* = 18)
Demographic characteristics	Age (years)	52 (IQR 41.25–66)
	Sex (male)	8 (44.4%)
Tumor shape	Tumor form oval	12 (66.7%)
	Tumor form round	6 (33.3%)
	Tumor volume (cm^3^)	7.895 (IQR 6.565–11.04)
	Sharp	15 (83.3%)
	Blurred	3 (16.7%)
	Maximum size axial (mm)	28 (22.75–34)
Tumor location	Left orbit	14 (77.8%)
	Right orbit	4 (22.2%)
	Location mediocranial	6 (33.3%)
	Location laterocranial	8 (44.4%)
	Location laterocaudal	2 (11.1%)
	Location mediocaudal	2 (11.1%)
	Intraconal	6 (33.3%)
	Extraconal	12 (66.7%)
	Ventral	6 (33.3%)
	Dorsal	12 (66.7%)

*IQR* interquartile range

**Table 2:** MR imaging characteristics of solitary fibrous tumors of the orbit

Categories	Variables	Total (*n* = 18)
MRI findings	Homogeneity on T2 without fat-saturation	Homogeneous 7 (41.2%)
		Inhomogeneous 10 (58.8%), 1 missing
	Flow voids	17 (94.4%)
	T2-weighted imaging: tumor compared to retrobulbar fat	Hypointense: 18 (100%)
	T1-weighted imaging: tumor compared to retrobulbar fat	Hypointense: 18 (100%)
	T1-weighted imaging: tumor compared to muscle tissue	Isointense 17 (100%), 1 missing
	T2-weighted imaging: tumor compared to muscle tissue	Hypointense 1 (5.6%)
		Hyperintense 17 (94.4%)
	T2-weighted imaging: tumor compared to brain cortex	Hypointense 2 (11.1%)
		Hyperintense 7 (38.9%)
		Isointense 9 (50%)
	T1-weighted imaging: tumor compared to brain cortex	Hyperintense 3 (17.6%)
		Isointense 14 (82.4%)
		1 missing
	Contrast-enhanced T1-weighted imaging: contrast enhancement of tumor	Homogeneous 10 (55.6%)
		Inhomogeneous 8 (44.4%)
	Contrast-enhanced T1-weighted imaging: tumor compared to temporomesial brain cortex	Hyperintense 17 (94.4%)
		Isointense 1 (5.6%)
	Contrast uptake ratio	1.2 (IQR 0.7–1.7)

*IQR* interquartile range

### Signal Characteristics of SFT On T1-weighted Imaging

**Fig. 3:**
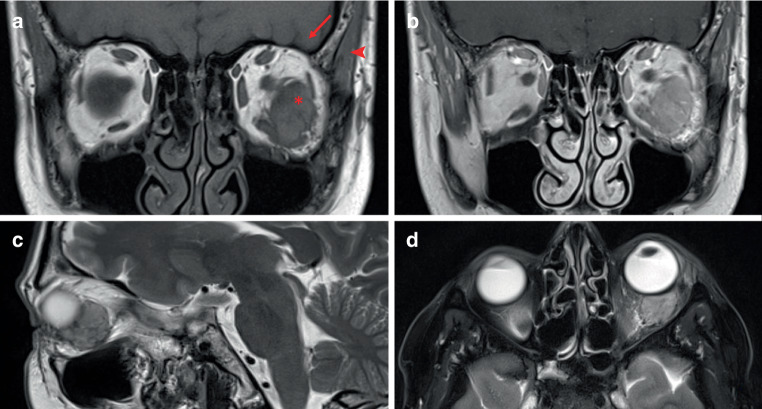
Magnetic resonance imaging of solitary fibrous tumor (SFT) in the laterocaudal quadrant and intraconal space of the left orbit in a 47-year-old woman. **a** coronal plane of non-contrast T1-weighted imaging showing signal isointensity between SFT (*red star*), brain cortex (*red arrow*) and temporal muscle (*red arrowhead*), **b** coronal plane of contrast-enhanced T1-weighted imaging illustrating the markedly increased contrast enhancement of SFT, **c** sagittal plane of T2-weighted imaging demonstrating slightly inhomogeneous tumor signal and the presence of flow voids, **d** axial plane of fat-saturated T2-weighted imaging showing similar signal intensity compared to brain cortex. *SFT* solitary fibrous tumor

On pre-contrast T1-weighted images, the SI of the tumor was significantly lower than that of the retrobulbar fat and appeared equivalent (82%) to the temporomesial brain cortex. Compared to the temporal muscle, the SI of the tumor was consistently isointense (Fig. 3).

### Signal Characteristics of SFT on T2-weighted Imaging

On T2-weighted images, the SI of the tumor was also lower than that of the retrobulbar fat. The tumors appeared isointense (50%) or slightly hyperintense (39%) when compared to the temporomesial brain cortex. Compared to muscle tissue, the SFTs were hyperintense in most cases (94%). The tumors showed significant signal heterogeneity, with inhomogeneous appearance in more than 50% of the cases.

### Contrast Uptake Behavior of SFT in Postcontrast T1-weighted Imaging

After contrast administration, the SFT appeared almost always hyperintense compared to the temporomesial brain cortex (94%). More than half of the lesions exhibited a homogeneous and intense contrast enhancement pattern (56%) characterized by a median SI of 2.2-fold compared to baseline precontrast T1-weighted imaging (mean uptake ratio 1.2, IQR 0.7–1.7).

## Discussion

Based on conventional MR imaging techniques, we evaluated the imaging characteristics of orbital SFTs in 18 patients. The SFTs of the orbit were typically oval in shape with a sharp margin. The most frequent location was the extraconal space in the cranial half of the orbit. On both T1-weighted and T2-weighted images the signal intensity of the SFT was consistently hypointense in comparison to the retrobulbar fat tissue, whereas it was isointense or slightly hyperintense relative to the temporal brain cortex. The SFTs were characterized by their hypervascularity, as the flow void phenomenon was present in almost all cases, and median tumor SI increased about 1.2‑fold after contrast uptake in over 50% of all cases.

The imaging features of orbital SFTs in our case series are in line with previously published case reports, in that they were commonly described as a well-defined and oval-shaped orbital mass with expanding features, predominantly extraconal, in the upper half of the orbit [10, 12]. Comparison of tumor SI with adjacent tissues such as retrobulbar fat, brain cortex, or muscles seems to be quite useful to differentiate SFT from other orbital tumors. Moreover, intense contrast enhancement and the presence of flow voids are key findings in SFT. The heterogeneous tumor signals seen on T2-weighted imaging in our study could be explained by the random distribution of collagenous tissue and sometimes the occurrence of cyst formation and hemorrhage [10].

The semi-malignant nature of SFTs is characterized by their potential malignant transformation, marked by histological features such as a high mitotic rate, increased cellularity, and pleomorphism, alongside the risk of recurrence and metastasis. Among the 18 patients in our study, 5 experienced recurrence (28%) after surgical resection. Our result was consistent with previous publications (recurrence ratio 28–37%) [13, 14]. Distant metastases were reported to occur in approximately 2% of cases [13]. Probably because of the limited number of patients, we did not find metastasis in our patient group. Therefore, imaging monitoring played a key role throughout the entire clinical course of SFT patients.

Due to the hypervascular nature of SFTs, accumulating data suggested the necessity of preoperative tumor embolization in selected cases to facilitate surgical procedures by minimizing intraoperative blood loss, reducing operative time, and inducing tumor shrinkage [15]. Large tumor size, extended hypervascularity, and a history of failed surgical removal attempts because of intraoperative bleeding were considered as rationales for decision-making of preoperative embolization [16, 17]. Additionally, challenging locations for complete surgical resection, such as the dorsal half of the orbit and proximity to the optic nerve, have also prompted the decision for preoperative embolization [15].

The following section compares the key imaging features of orbital SFT with other orbital tumors, including cavernous hemangioma (or cavernous vascular malformation, CVM), orbital lymphoma, inflammatory pseudotumor, and schwannoma.

Consisting of dilated blood vessels, CVMs are classified as the most common orbital mass lesion in adults [18]. The estimated incidence is 0.15–0.56 per 100,000 persons per year [19]. Similar to SFTs, CVMs usually have well-defined margins and an oval shape; however, unlike SFTs, the most frequent location of CVMs is the intraconal space [20]. Furthermore, CVMs appear much more hypointense than extraocular muscle tissue on T1-weighted imaging due to their higher content of blood volume, whereas SI is markedly high on T2-weighted images. The CVMs often demonstrate a progressive enhancement pattern, starting with a small point or area of enhancement and ending with homogeneous enhancement of the tumor [20].

Orbital lymphoma represents a small fraction (approximately 2%) of all systemic non-Hodgkin lymphomas [21]. It consists of a heterogeneous group of malignancies and accounts for approximately 20% of all primary tumors of the orbit [22]. Lymphomas can involve both intraorbital and extraorbital regions, most frequently in the extraconal space [23]; however, they have a higher tendency to affect the preseptal space [22], which was not seen in any of the SFT patients in our study. Other differences include the less frequent presence of flow voids and the characteristic homogeneous enhancement pattern of lymphoma [24]. Due to the higher cellularity of lymphoma, MR functional parameters like apparent diffusion coefficient maps may play a crucial role in further differentiating lymphoma from other orbital mass lesions [24].

Idiopathic orbital inflammation (or inflammatory pseudotumors) is a poorly marginated and mass-like enhancing inflammatory tissue that may involve intraorbital or extraorbital regions. It accounts for 8–10% of all orbital disorders [25]. The dacryoadenitis and myositis forms have been reported as the most frequent disease patterns. Inflammatory pseudotumors were variably hypointense on T1-weighted images. Compared with the brain cortex or extraocular muscles, they were mainly hypointense to isointense on T2-weighted images due to cellular infiltrates and fibrosis, in contrast to SFTs [26]. Inflammatory pseudotumors may exhibit marked contrast enhancement [22].

Orbital schwannomas are rare, accounting for approximately 1% of orbital neoplasms [27]. They usually show a well-defined margin and are often associated with one or more small cysts, adding to their unique appearance. On T1-weighted images, schwannomas exhibit either uniformly reduced hypointensity or hypointensity in the center with isointensity/ or hyperintensity in the periphery. On T2-weighted MR images, the central areas of schwannomas are often relatively hyperintense with peritumoral isointensity or hypointensity. Schwannomas frequently display homogeneous enhancement patterns, while a peripheral ring enhancement has also been reported in more than 25% of cases [28].

Advanced MR imaging techniques like dynamic contrast-enhanced MRI (DCE-MRI), time-resolved angiography with stochastic trajectories (TWIST) and intravoxel incoherent motion (IVIM) DWI might serve as promising imaging tools in delineating the microvascular and macrovascular features of hypervascular tumors [29, 30]. Thus, implementation of such advanced imaging approaches may further increase the diagnostic certainty to distinguish SFTs from other orbital tumors. Initial results of DCE-MRI have shown to be promising in the differentiation between schwannoma and orbital SFTs [8, 31].

Our study has several limitations. First, it is a retrospective analysis with only 18 patients. The diagnostic value of the imaging findings is moderately conclusive due to tumor variability. Second, the available MR images of our patient collective covered a broad range of 20 years, resulting in inconsistent imaging quality and interpretation. Third, advanced MR imaging techniques such as dynamic contrast-enhanced imaging, diffusion-weighted imaging, and radiomics with data segmentation algorithms were not available in our study.

Based on conventional MR techniques, the imaging characteristics of orbital SFTs found in our study coupled with a comprehensive understanding of potential differential diagnoses, empower a more precise differentiation between orbital SFTs and other common orbital mass lesions.

## Conclusion

Solitary fibrous tumors of the orbit are rare and diagnostically challenging. They were typically oval shaped with a well-defined margin. Extraconal space and cranial half of the orbit were found as the most frequent tumor locations. They were characterized by typical flow voids phenomenon and significant contrast enhancement due to their hypervascular nature. Comparison of tumor signal intensity with adjacent tissues may further improve distinguishing SFTs from other orbital tumor entities.
